# IL-23/Th17 pathway and *IL-17A* gene polymorphism in Egyptian children with immune thrombocbytopenic purpura

**DOI:** 10.1186/s13052-021-01131-3

**Published:** 2021-08-26

**Authors:** Ahlam M. Ismail, Aliaa M. Higazi, Hanan M. Nomeir, Naglaa M. Farag

**Affiliations:** 1 Minia Maternity and Children University Hospital, Pediatrics department, Faculty of Medicine, Minia University, Minia, Egypt; 2 Clinical and Chemical Pathology department, Faculty of Medicine, Minia University, Minia, Egypt; 3 Medical Biochemistry department, Faculty of Medicine, Alexandria University, Alexandria, Egypt

**Keywords:** IL-23, Th17, IL-17A, Polymorphism, ITP, Children

## Abstract

**Background:**

Immune thrombocytopenic purpura (ITP) is an acquired complex autoimmune thrombocytopenia. Uncontrolled cellular immune response is one of the key triggers for the loss of immune tolerance in ITP patients. The purpose of this study was to investigate the association of IL-23/Th17, IL-17A and *IL-17A* rs2275913 gene polymorphism with ITP in Egyptian children.

**Methods:**

60 patients with ITP and 50 healthy control children from Minia city- Egypt were involved. Serum levels of IL-23 and IL-17A were determined by enzyme-linked immunosorbent assay. The frequency of Th17 cells was measured using flow cytometer. Genotyping for *IL-17A* was performed via polymerase chain reaction-restriction fragment length polymorphism.

**Results:**

Comparing children with ITP to controls, serum levels of IL-23 and IL-17A as well as Th17 cells percentage were significantly increased (*p* <  0.001). Also, higher levels of these ILs and Th17 cells percentage were associated with decreased platelet count within ITP patients (p <  0.001). Analysis of genotype frequencies for *IL-17A* rs2275913 polymorphism and its alleles (A, G) showed no significant difference between cases and controls. Likewise, no significant differences were demonstrated between acute and chronic ITP regarding both *IL-17A* rs2275913 polymorphism prevalence and levels of IL-23, IL-17A plus Th17 cells percentage. The frequency of A alleles was 85 and 86% within patients and controls, respectively.

**Conclusions:**

Elevated levels of IL-23, IL-17A and Th17 cells may be involved in ITP pathogenesis while *IL-17A* polymorphism rs2275913 is not prevalent in Egyptian children with ITP.

## Background

Immune thrombocytopenic purpura (ITP) is a common acquired autoimmune thrombocytopenic syndrome in children. It is characterized by immune-mediated platelet destruction due to binding of immunoglobulin (Ig) G autoantibodies with platelet glycoproteins (GPs) mostly GPIIb/IIIa and GPIb/IX [1]. The incidence of ITP is approximately 12 per 100,000 children per year and its associated mortality rate is about 1–3% per year in severe cases [2, 3].

The breakdown in the immune tolerance mechanism with ITP is mainly due to dysregulation in their cellular immune responses [4]. ITP patients have platelet autoreactive B cells but T cells immune abnormalities and cytokine imbalance are involved as well [5]. In this way, excessive stimulation of cytotoxic T-lymphocytes induces autologous platelets destruction [6]. Also, the increased apoptosis of megakaryocytes thus decreased platelet production mediated by CD8^+^ T cells has been linked to ITP [7]. An additional mechanism for loss of peripheral immune tolerance is defective suppressive function or decreased number of a distinct subpopulation of CD4^+^ T cells called regulatory T cells (Treg) [7]. Moreover, uncontrolled T-helper (Th) cells play crucial roles in the pathogenesis of ITP [7, 8].

Th17 cells are regarded as promoters of autoimmune conditions as they produce a number of pro-inflammatory cytokines containing IL-17A accordingly induce tissue damage [9]. IL-17A is a member of a cytokine family of six cytokines: IL-17A-F. IL-17A uses the IL-17 receptor A (IL-17RA)-IL-17RC heterodimer for signaling [10]. IL-17RA is expressed on hematopoietic cells at high levels [11]. This has led to an interest in using IL-17A as therapeutic target in hematologic disorders [12]. The imbalance of Th17/Treg toward Th17 cells has been shown to play an important role in the peripheral immune response [13, 14]. IL-23 is crucial for maintenance and expansion of Th17 [15]. Releasing IL-17A in response to IL-23/Th17 targets mainly mesenchymal and myeloid cells. It could promote the production of a variety of pro-inflammatory or hematopoietic cytokines, chemokines, matrix metalloproteinases plus effecting the expansion of neutrophil through granulocyte colony-stimulating factor (G-CSF) and chemotaxis (CXC), thus aggravating the immunologic derangement characteristic of ITP [16].

Previous studies showed that IL-23/Th17 pathway and increased expression of *IL-17A* as well as *IL-17A* gene polymorphism have been associated with various autoimmune diseases [17], such as primary biliary cirrhosis [18], inflammatory bowel disease [19, 20], rheumatoid arthritis [21–23] and ulcerative colitis [21, 24]. However, the role of Th17 and IL-23 in the pathogenesis of ITP was only reported in limited number of studies [25, 26]. Furthermore, the significances of IL-17A and its genetic variations in children with ITP remain uncertain particularly when referred to distinct/regional population groups like Egyptian children [27].

In the present study, we estimated the frequency of Th17 cells in addition to serum levels of both IL-23 and IL-17A in children with primary ITP compared to healthy controls. On top, we compared their measurements between children with acute and chronic ITP. The association between *IL-17A* rs2275913 gene polymorphisms and ITP susceptibility, its chronicity along with severity was evaluated as well.

## Methods

### Subjects and clinical data collection

The current study is a case control one that was conducted in the Hematology Unit, Department of Pediatrics, Minia Maternity and Children University Hospital, Egypt. A total of 110 children were included, 60 children with primary ITP and 50 age and sex matched apparently healthy children as a control group. After obtaining clearance from the Faculty of Medicine, Minia University ethical committee, a written informed consent was signed from the parents or guardians of the children.

Patients were diagnosed based on the presence of bruising and/or petechiae or mucous membrane bleeding, platelet count < 100 ×  10^9^/L and increased or normal count of megakaryocytes in bone marrow aspirates [28]. Children with active infection, splenomegaly, lymphadenopathy, and any other underlying diseases which may cause thrombocytopenia were excluded from the study. We also excluded infants aged less than 6 months.

ITP patients were receiving a treatment protocol according to guidelines of the American Society of Hematology 2019 for immune thrombocytopenia [28]. Follow-up of patients was carried out to detect remission or progression of the persistent or chronic course of the disease. Three forms of ITP are distinguished according to duration of disease from the onset of the illness: acute < 3 months, persistent 3–12 months, and chronic > 12 months. ITP severity had classified according to platelets count into very severe = platelet count < 10 × 10^9^/L; severe = platelet count 10–30 × 10^9^/L; moderate = platelet count 30–50 × 10^9^/L, and mild = platelet count > 50 × 10^9^/L. [28]

All enrolled children underwent detailed history taking and clinical examination plus routine investigations. As well, laboratory workup was performed to all subjects including complete blood count using automated cell counter Sysmex diagnostic, USA. Also, examination of Leishman-stained peripheral blood smears and bone marrow aspiration smears were carried out. Our Hematology Unit protocol recommend bone marrow aspiration to all suspected ITP patients to rule out leukemia, myelodysplastic syndrome, or aplastic anemia especially because some of our ITP patients presented with anemia beside thrombocytopenia. Additionally, we use steroids as first line therapy due to the cost price of intravenous immunoglobulin (IVIG) [29].

### Laboratory investigations

Human IL-23 enzyme-linked immunosorbent assay (ELISA) Kit (abcam, United Kingdom) and human IL-17A ELISA Kit (Quantikine, Bio-Techne Ltd) were used for quantitative measurement of IL-23 and IL-17A serum levels; respectively. The detection protocols were performed according to the manufacturers’ instructions. Levels of ILs were expressed as pg/mL.

Flow cytometer (BD Biosciences, USA) was used to detect the frequency of Th17 cells. CD3^+^CD4^+^ cells positive for IL-17 were recognized as Th17 cells. Heparinized whole blood was surface stained with PE/Cy7-labeled monoclonal anti-CD4 and APC-labeled monoclonal anti-CD3 antibodies (BD Biosciences, USA). Then, red blood cells were lysed using FACS lysing buffer (BD Biosciences, USA). Afterwards, cells were washed with phosphate buffered saline (PBS, pH 7.2, 0.15 M), fixed with a fixation buffer from Leucoperm, ABD Serotech, USA followed by washing again for 2 times with PBS. The cells were later permeabilized with permeabilization buffer (Leucoperm, ABD Serotech, USA) and stained with phycoerythrin-(PE) labeled monoclonal antibodies against IL-17A. Subsequently, flow cytometry analysis was performed in CD3^+^ lymphocytes gate [30].

*IL-17A* rs2275913 polymorphism genotyping was detected by the polymerase chain reaction-restriction fragment length polymorphism (PCR-RFLP) technique. DNA was extracted from whole blood using QIAamp DNA blood Mini Kit from Qiagen Inc., Germany. The following primers were used; forward 5′-TCT CCA TCT CCA TCA CCT TTG-3’and reverse 5′-GTC CAA ATC AGC AAG AGC ATC-3′. PCR products were digested using *XagI* (New England BioLabs, England) *and d*igested amplicons were separated via electrophoresis with agarose gel (2%) [27].

### Statistical analysis

The analysis of the data was carried out using the IBM SPSS 20.0 statistical package software. Normality of the data was tested using the Shapiro-Wilk or Kolmogorov-Smirnov tests. Data were expressed as mean ± standard deviation (SD), minimum and maximum of range for quantitative parametric measures or median and range for quantitative non-parametric measures in addition to both number and percentage for categorized data. The Student t-test was used for comparison between two independent groups for parametric data and Mann-Whitney test for non-parametric data. For comparison between more than two independent groups, analysis of variance (ANOVA) was used for comparison for parametric data. Chi-square test or Fisher’s exact test were used to compare categorical variables. Correlations between the parameters were analyzed by Pearson’s correlation analysis. Correlation coefficient was considered weak if r = 0–0.24, fair if r = 0.25–0.49, moderate if r = 0.5–0.74 and strong if r = 0.75–1. A *p*-value less than 0.05 was considered significant.

## Results

### Demographic and laboratory data of studied groups

The study included 60 children with ITP (male/female, 38/22; Age range 1–12 years) attending our Pediatric Hematology Unit and 50 healthy controls (male/female, 24/26; Age range 1–14 years). Platelet count in ITP patients ranged from 9 × 10^9^/L-67 × 10^9^/L, with median platelet count of 22 × 10^9^/L at initial diagnosis. Demographic and laboratory data of all patients and healthy controls are shown in (Table 1). Compared to healthy controls, statistically significant lower platelet count and hemoglobin concentration along with higher Mean platelet volume (MPV), Th17 cells percentage, and serum levels of IL-23 and IL-17A were detected in ITP patients **(**Table 1**).** Regarding Th17, IL-23 and IL-17A, we found no statistically significant difference in their levels in chronic ITP patients when compared with those with acute ITP **(**Table 2**)**.

**Table 1:** Demographic and laboratory data of patients and controls

Parameter	ITP patients (*N* = 60)	Control (*N* = 50)	*P*-value
Age (year)			0.345
Range	1–12	1–14	
Mean ± SD	5·5 ± 3·2	6·8 ± 3·9	
Sex			0.254
Male	38 (63.3%)	24 (48.0%)	
Female	22 (36.7%)	26 (52.0%)	
Weight centile ^(th)^			0.09
Range	75–90	75–80	
Mean ± SD	49.8 ± 20.8	47.8 ± 18.5	
Length/Heigh centile ^(th)^			0.07
Range	50–75	50–75	
Mean ± SD	64.8 ± 11.6	61.24 ± 12.4	
Residence			0.234
Rural	32 (53.0%)	28 (56.0%)	
Urban	28 (47.0%)	22 (44.0%)	
Hb (gm/dL)			< 0.001*
Range	7–11.5	11–12.4	
Mean ± SD	9.1 ± 1.2	11.6 ± 0.4	
WBCs × 10^9^/L			0.354
Range	4–13	4.8–13.6	
Mean ± SD	6.8 ± 2	7.3 ± 2.7	
Platelets × 10^9^/L			< 0.001*
Range	9–67	218–461	
Mean ± SD	52.9 ± 25.6	280.3 ± 46.4	
MPV, fl			0.006*
Range	9.4–11.4	7.7–8.9	
Mean ± SD	11.2 ± 1.6	7.9 ± 5	
Th17 (%)			< 0.001*
Range	1–3.9	0.3–1.9	
Mean ± SD	2.1 ± 0.9	1.1 ± 0.4	
IL-23 (pg/mL)			< 0.001*
Range	109.6–378.6	34.9–140.7	
Mean ± SD	215.4 ± 79	86.4 ± 32.7	
IL-17A (pg/mL)			< 0.001*
Range	40.7–369	31–191.9	
Mean ± SD	92.4 ± 39	42.7 ± 19	

*N* number, *SD* standard deviation, *Hb* hemoglobin, *WBCs* white blood cells, *MPV* mean platelets volume

*p-value < 0.05 = significant

**Table 2:** Laboratory data of acute and chronic ITP patients

Parameter	ITP		*p*-value
	Acute (< 3 m) (*N* = 26)	Chronic (> 12 m) (*N* = 28)	
	Median (range)	Median (range)	
**Hb (gm/dL)**	9 (8–11)	9 (7–11.5)	0.713
**WBCs (× 10**^**9**^**/L)**	6.2 (4–9.7)	6 (4.5–9)	0.593
**PLTs (× 10**^**9**^**/L)**	19 (9–55)	23 (11–67)	0.789
**Th17 (%)**	1.7 (1–3.6)	2.1 (1–3.6)	0.467
**IL-23 (pg/mL)**	183.6 (110.3–345.7)	219 (109.6–353.3)	0.662
**IL-17A (pg/mL)**	82.1 (42.3–191.9)	117.6 (40.7–173.9)	0.961

*N* number, *Hb* hemoglobin, *WBCs* white blood cells, *PLTs* platelets

### Presenting symptoms and therapeutic regimens of included ITP children

50% of patients had prior viral infections and 80% had cutaneous bleeding in the form of purpura or ecchymosis. Bleeding per gums or epistaxis were presented in 36.7% of patients. Intracranial hemorrhage occurred in two chronic ITP patients (3.3%) who had very severe thrombocytopenia.

Patients with ITP were treated as recommender per the American Society of Hematology 2019 guidelines [28]. In the current study, 52 children (86.7%) received steroids, 10 patients (16.7%) received IVIG and 8 children with chronic ITP (13.3%) treated with azathioprine (Immuran). We added eltrombopag (Revolade) to 18 children with chronic ITP (30%). Four chronic patients underwent splenectomy (6.7%).

### Correlation between IL-23, IL-17A, Th17 cells and platelet count in ITP group

**Fig. 1:**
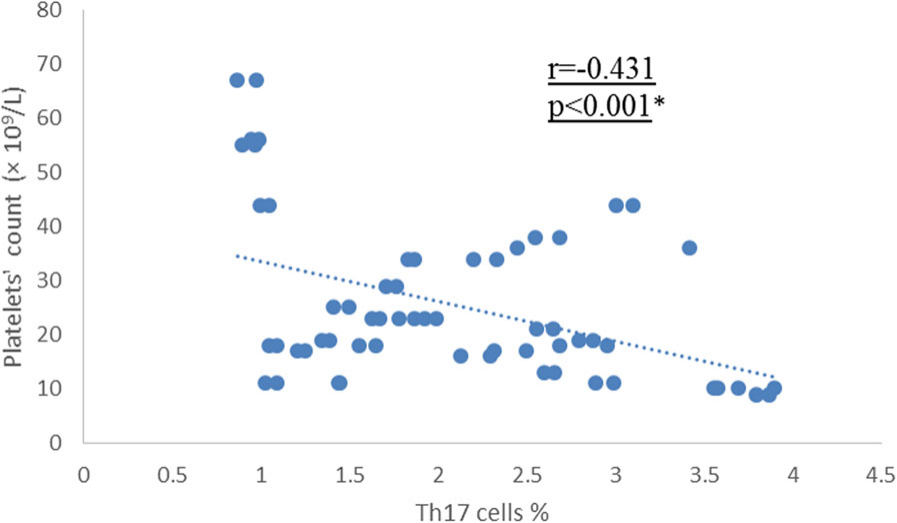
Correlation between Th17 cells percentage and platelet count within ITP patients. There was statistically significant fair negative correlation between Th17 cells percentage and platelet count in patients’ group (r = −0.431 & *p* < 0.001). *p-value < 0.05 = significant

Table 3 shows that IL-23 levels have significant positive correlation with IL-17A levels and Th17 cells percentage in ITP cases. Similarly, IL-17A is shown to be positively correlated with Th17 cells percentage in patients’ group (*p* <  0.001) **(**Table 3**)**. Though, negative correlations are shown between platelet count and both IL-23 and IL-17A within ITP patients **(**Table 3**)**. Figure 1 shows the fair statistically significant negative correlation between Th17 cells percentage and platelets’ count in ITP patients’ group (r = − 0.431 & *p* <  0.001).

**Table 3:** Correlation between IL-23, *IL-17A*, Th17 and platelet count within ITP group

Parameter	IL-23 (pg/mL)		IL-17A (pg/mL)	
	r	*p*	r	*p*
IL-17A (pg/mL)	0.744	< 0.001*	–	–
Th17 (%)	0.987	< 0.001*	0.758	< 0.001*
Platelet count	−0.393	< 0.001*	−0.210	< 0.001*

*p-value < 0.05 = significant

Table 4 shows the genotype and allele frequency of *IL-17A* rs2275913 polymorphism in ITP cases**.** In ITP patients, the genotype frequencies of *IL-17A* rs2275913 polymorphism were AA (76.7%), AG (16.7%), and GG (6.6%). While the genotype frequencies of *IL-17A* rs2275913 polymorphism in the control group, were AA (72%) and AG (28%). There were no statistically significant differences between frequencies of the three *IL-17A* rs2275913 genotypes in ITP patients compared to control group. In addition, although *IL-17A* rs2275913 GG genotype was only found in cases, the difference of genotype frequency between patients and normal controls did not reach statistic difference. The frequency of allele A of *IL-17A* rs2275913 was 85% in the cases group and 86% in the control group. Similarly, the frequency of allele G was 15% in the cases group and 14% in the control group.

**Table 4:** Genotype frequency of *IL-17A* gene in patients and control

Parameter	ITP	Control	OR (95% CI)	*p*-value
*IL-17A* genotype	N (%)	N (%)		
AA	46 (76.7%)	36 (72.0%)	1.28 (0.38–4.31)	0.639
AG	10 (16.7%)	14 (28.0%)	0.51 (0.14–1.88)	0.315
GG	4 (6.6%)	0 (0.0%)	NA	NA
A alleles G alleles	102 (85.0%) 18(15.0%)	86 (86.0%) 14 (14.0%)	–	–

*N* number, *CI* confidence interval

*NA* non applicable

Table 5 shows the genotype and allele distribution among three ITP subgroups. These subgroups were subdivided according to the course of ITP into acute, persistent, and chronic ITP. No statistically significant differences were found when these subgroups were compared to each other. In addition, we investigated the association of the *IL-17A* rs2275913 genotypes with some clinical features of ITP. There was no significant association between the frequencies of the genotypes and gender, age, or degree of ITP severity **(**Table 6**)**.

**Table 5:** Relation between outcome of ITP and *IL-17A* genotypes

Parameter	Acute ITP (< 3 m) *N* = 26	Persistent ITP (3-12 m) *N* = 6	Chronic ITP (> 12 m) *N* = 28	*p*-value
	N (%)	N (%)	N (%)	
**AA**	22 (84.6%)	4 (66.7%)	20 (71.4%)	0.578
**AG**	4 (15.4%)	2 (33.3%)	4 (14.3%)	0.618
**GG**	0 (0.0%)	0 (0.0%)	4 (14.3%)	NA

*N* number, *NA* non applicable

**Table 6:** Relation of age, sex, and degree of thrombocytopenia with *IL-17A* genotypes

Parameter	*IL-17A* Genotype			*p*-value
	AA	AG	GG	
	*N* = 46	*N* = 10	*N* = 4	
**Age (years)**				0.266
Mean ± SD	6.7 ± 3.5	7 ± 3.7	11 ± 1.4	
(Range)	[2–12]	[1–10]	[10–12]	
**Sex**				
Male	28 (60.9%)	6 (60.0%)	4 (100.0%)	0.820
Female	18 (39.1%)	4 (40.0%)	0 (0.0%)	
**Thrombocytopenia**				
Very severe	0 (0.0%)	2 (20.0%)	0 (0.0%)	
Severe	28 (60.9%)	8 (80.0%)	4 (100.0%)	0.380
Moderate	12 (26.1%)	0 (0.0%)	0 (0.0%)	
Mild	6 (13.0%)	0 (0.0%)	0 (0.0%)	

*N* number, *SD* standard deviation

## Discussion

Our study included 110 children: 60 children with ITP and 50 healthy controls. There were statistically significant lower platelet count and hemoglobin concertation in ITP patients compared to healthy controls which agrees with the results of El Husseiny et al. 2018 [31]. They found statistically significant lower platelet count along with hemoglobin levels in cases rather than controls. Decreased hemoglobin concentrations in ITP patients can be due to nutritional iron deficiency anemia and/or blood loss because of frequent bleeding attacks.

Moreover, our results showed significant increase in MPV in ITP children when compared to control group which is similar to data in the study done by Baig 2015 [32] who found significant increase in MPV within ITP patients and recommended the use of platelet indices as a diagnostic tool to differentiate ITP from acute leukemias.

T-cell is important in the pathogenesis of ITP [33]. The Th17 cell, which produces IL-17, is a subset of T helper cells. Th17 cells play an important role in T-cell-mediated diseases hence may play a role in ITP [34]. As a classical autoimmune disorder, ITP has also been reported to have an aberrant Th17 profile [35].

Higher Th17 cells percentage were detected in the present study within ITP patients in comparison to healthy controls. This comes in agreement with Rocha et al. 2013 [36], who reported that Th17 cells were significantly higher in active ITP patients than that in healthy controls. Likewise, the study of Zhang et al. 2009 [37] showed increased levels of circulating Th17 cells in children with chronic ITP compared to healthy controls. Moreover, we detected higher serum levels of IL-23 in ITP children than controls**.** This comes in harmony with the results of Ye et al. 2015 [25], who also found increased levels of IL-23 in patients with primary ITP compared to healthy controls. They concluded that IL-23/Th17 pathway may be involved in the pathogenesis of ITP through enhancement of the Th17 response.

Th17 cells and their characteristic IL-17A cytokine are considered the initiators of various autoimmune conditions [9]. This has led to an interest in using them as therapeutic targets in these disorders [12]. The expression of *IL-17A* gene can be upregulated by stimulation with potent IL-23 leading to Th17 differentiation and active release of IL-17 family of cytokines in T lineage lymphocytes [38].

Higher IL-17A levels in ITP children than controls were found in our study. This comes in concordance with the results of El Husseiny et al. 2018 study [31]. They reported higher expression of IL-17 and explained this by the effect of using immune suppressors or upregulation of their receptors on Treg cells which have resistance to their activity. In contrast, Hunag et al. 2015 [39] found that IL-17 levels are indifferent between chronic ITP and normal controls. Different population and group of patients may explain this contradictory.

Furthermore, our results revealed that IL-23 level has significant positive correlation with IL-17A levels and Th17 cells percentage in ITP cases besides serum levels of IL-17A is positively correlated with the percentage of Th17 cells in patients as well. This positive correlation can suggest that IL-17 production is influenced by IL-23. Reversely, negative correlation was found between IL-23, Th17 cells percentage along with IL-17A and platelets count in ITP patients. This comes in line with El Husseiny et al. 2018 [31] and Ye et al. 2015 [25] studies. They found positive correlations between IL-23 levels along with levels of IL-17 and Th17 cells percentage in ITP patients, while negatively related to platelet counts. They suggested that the platelet counts reflect the disease development as well as its severity thus; IL-23 levels could serve as a potential index to evaluate the disease state. In contrast, El Husseiny et al. 2018 [31] found statistically insignificant correlation between platelets count and the level of IL-17 in ITP cases.

In ITP patients, the genotype frequencies of the *IL-17A* rs2275913 polymorphism were AA (76.7%), AG (16.7%) and GG were found in (6.6%) while, in the control group the genotype frequencies of the *IL-17A* rs2275913 polymorphism, were AA (72%) and AG (28%). There was no significant difference between frequencies of the three *IL-17A* genotypes and alleles distribution in ITP patients compared to control group despite *IL-17A* GG genotype was only found in cases. This comes in concordance with the study of Liu et al. 2016 [40].

The frequency of allele A of *IL-17A* gene was 85% in the cases group and 86% in the control group, while the frequency of allele G was 15% in the cases group and 14% in the control group which were not significantly different when cases compared to controls. Similarly, Aziz et al. 2018 [27] carried out statistical analysis of the genotype frequencies (GG, AG, AA) of the *IL-17A* rs2275913 polymorphism and its alleles (A, G) in cases with acute ITP compared to controls and found no significant differences between the two groups. Aziz et al., 2018 study [27] was performed on Egyptian children. The frequencies of A allele were 71.3 and 75.5% when ITP cases were compared to controls; respectively. Other studies were performed on Egyptian population regarding A allele frequency of *IL-17A* rs2275913 polymorphism in Egyptian population. The targeted patients in these studies were those with systemic lupus erythematosus [41], acute myeloid leukemia [42] and vitiligo [43]. Similar to our study and Aziz et al., 2018 study [27], their reported frequencies were 65% within healthy control group in the study concerned about acute myeloid leukemia patients and 53.8% within healthy control group in vitiligo concerned one. In contrast, A allele frequency of *IL-17A* rs2275913 gene shows a minor percentage (29%) within healthy controls in systemic lupus erythematosus study which comes in consistent with *IL-17A* rs2275913 polymorphism allele frequencies that are reported in SNP public database (https://www.ncbi.nlm.nih.gov/snp/rs2275913#frequency_tab). Altogether, these findings indicate that North Africans may have different *IL-17A* rs2275913 polymorphism characteristics, particularly Egyptians. Therefore, additional Egyptian population data base collection about *IL-17A* rs2275913 SNP is necessary.

On top, we investigated the association between *IL-17A* rs2275913 polymorphism and the clinical responses to treatment. Steroid treatment is the first-line therapy strategy, thus most of our enrolled patients (86.7%) received it. We found statistically insignificant difference with the outcome of treatment. Regarding the disease course and prognosis of ITP patients during their follow-up, we evaluated the genotype and allele distribution among three ITP subgroups: acute, persistent, and chronic ITP. Statistically insignificant differences were found with comparison between them. IL-17A AA genotype appeared to be more closely associated with early recovery than with persistent course of the disease (84.6% vs. 66.7%) and AG genotype more closely associated with persistent disease course (33.3% vs. 15.4%). Our results come in line with those of Aziz et al. 2018 [27]. They found that the IL-17A rs2275913 GG genotype was associated with early recovery (*p* = 0.04). Also, we investigated the association of IL-17A genotypes with the clinical features of acute ITP. There was no significant association between the frequencies of genotypes and gender, age as well as disease severity. Likewise, Aziz et al. 2018 [27] investigated the association of the *IL-17A* rs2275913 genotypes with the clinical features of acute ITP. They found no significant association between genotypes frequencies and gender, age, along with disease severity, or the outcome following treatment except for GG genotype which was expressed only in males and associated with severe thrombocytopenia (100%).

To the extent of our knowledge, our study is one of very few studies concerned about IL-23/Th17 plus IL-17A and its rs2275913 polymorphism in Egyptian children with ITP. However, this study was conducted on a relatively small number of ITP children recruited from one hospital. Among IL-17 family of cytokines, only *IL-17A* rs2275913 polymorphism was analyzed in the present study because it was reported as the most common *IL-17* gene polymorphism within ITP patients. Therefore, future studies on multicenter larger sample size Egyptian population are still warranted. These studies should focus on IL-23/Th17 and IL-17 in addition to different genes polymorphisms related to IL-17 family with before and after effective treatment evaluations.

## Conclusion

A higher percentage of Th17 cells along with elevated levels of IL-23 and IL-17A in ITP patients compared to healthy controls, as well as the significant positive correlation of IL-23 levels with IL-17A levels and Th17 cells percentage plus its significant negative correlation with platelet count suggest that IL-23 may be involved in the pathogenesis of ITP. However, this needs further comprehensive mechanistic investigations. These findings also provide new insights on the possibility of employing anti-IL-23 drugs or targeting the IL-17 axis for treatment of ITP which should be confirmed through animal models or in vitro trials. Moreover, no significant association was demonstrated in the current study between *IL-17A* rs2275913 polymorphism and the risk of ITP in Egyptian children which in turn requires prospective studies.

## Data Availability

Key data generated or analyzed during this study are included in this published article.

## Abbreviations

ITP: Immune thrombocytopenic purpura
IL-17A: interleukin-17A
IL-23: interleukin-23
Th17: T helper 17
GP: platelet glycoprotein
CD8, 4, 3: Cluster of differentiation 8, 4, 3
Treg: regulatory T cells
IL-17R: IL-17 receptor
G-CSF: granulocyte colony-stimulating factor
CXC: chemotaxis
IVIG: intravenous immunoglobulin
ELISA: enzyme-linked immunosorbent assay
PCR-RFLP: polymerase chain reaction-restriction fragment length polymorphism
SD: standard deviation
ANOVA: analysis of variance
MPV: Mean platelet volume
SNP: single nucleotide polymorphism
